# Association Between Autoimmune Thyroiditis and Cervical Artery Dissection: A Retrospective Cohort Study

**DOI:** 10.1002/hsr2.72161

**Published:** 2026-03-22

**Authors:** Robert J. Trager, Pratheek S. Makineni, Debbie S. Wright

**Affiliations:** ^1^ Connor Whole Health University Hospitals Cleveland Medical Center Cleveland Ohio USA; ^2^ Department of Family Medicine and Community Health Case Western Reserve University School of Medicine Cleveland Ohio USA; ^3^ Department of Biostatistics and Bioinformatics Clinical Research Training Program Duke University School of Medicine Durham North Carolina USA; ^4^ Case Western Reserve University School of Medicine Cleveland Ohio USA; ^5^ Parker University Dallas Texas USA; ^6^ Private practice, The Grove Health and Wellness Courtenay British Columbia Canada

**Keywords:** autoimmune diseases, cardiovascular diseases, cerebrovascular disorders, internal carotid artery dissection, thyroid diseases, vertebral artery dissection

## Abstract

**Background and Aims:**

Limited evidence suggests autoimmune thyroiditis (AT) could represent a risk factor for cervical artery dissection (CeAD). We tested the hypothesis of a positive association between AT and CeAD within 3 years following diagnosis compared to matched euthyroid controls.

**Methods:**

We searched a de‐identified United States' data resource (TriNetX LLC) for adults age ≥ 18 with newly‐diagnosed AT, and euthyroid controls, from 2014 to 2024. Propensity matching controlled for variables associated with CeAD. Our primary outcome was the risk ratio (RR) for CeAD within 3 years' follow‐up. Secondary outcomes explored the cumulative incidence of CeAD and RRs for vertebral and carotid artery dissection and stroke.

**Results:**

After matching, there were 143,831 patients per cohort (80% female). Comparing the AT cohort to matched euthyroid controls, the incidence and risk of CeAD was significantly greater [95% CI] (0.040% vs. 0.025%; RR = 1.58 [1.04, 2.40]; *p* = 0.029). Secondary outcomes included a non‐significant increase in incidence and risk of vertebral artery dissection (0.022% vs. 0.017%; RR = 1.28 [0.76, 2.16]; *p* = 0.354), and significant increase in incidence and risk of carotid artery dissection (0.019% vs. 0.008%; RR = 2.33 [1.19, 4.59]; *p* = 0.011) and stroke (2.1% vs. 1.3%; RR = 1.67 [1.58, 1.77]; *p* < 0.001). Cumulative incidence of CeAD in patients with AT highlighted a time‐dependent increase relative to euthyroid controls.

**Conclusion:**

The present findings suggest that AT is a risk factor for CeAD. Additional research is needed to corroborate these findings and clarify factors that may mediate the AT‐CeAD association, such as medications, thyroid hormones, and antibody levels.

## 1 Introduction

Cervical artery dissection (CeAD) is a serious condition characterized by a tear in the wall of a cervical artery, leading to an intramural hematoma and potentially causing a stroke [1]. The incidence of CeAD is approximately 8.9 per 100,000 person‐years [2]. Autoimmune thyroiditis (AT), also called Hashimoto's thyroiditis, represents the most common cause of hypothyroidism in regions with adequate iodine intake, such as the United States (US) [3, 4]. AT is an established risk factor for stroke [5] and is associated with increased risk of several cardiovascular diseases [6, 7, 8, 9], including aortic dissection and spontaneous coronary artery dissection [10, 11]. However, there is limited research regarding whether AT is a risk factor for CeAD [12, 13].

While recognized causes of CeAD include trauma and/or iatrogenic arterial injury related to surgery [14, 15], the underlying risk factors for non‐traumatic or spontaneous CeAD are less understood. Research points to several risk factors, including fibromuscular dysplasia, systemic lupus erythematosus, and other connective tissue disorders [14, 16, 17], hypertension [18, 19], migraine [20], pregnancy [21, 22], and respiratory infections [23, 24, 25, 26]. However, an emerging hypothesis proposes that AT may represent a novel CeAD risk factor due to its shared immunologic mechanisms with other conditions already established as CeAD risk factors. Chiefly, this hypothesis proposes that anti‐thyroid antibodies may cross‐react with proteins present in connective tissue of the cervical arteries, which could contribute to arterial wall vulnerability [13, 27]. While AT is relatively common in the general population, making it an ideal autoimmune condition to examine with respect to potential CeAD risk, no large epidemiological study has been conducted to date. The potential association between AT and CeAD remains insufficiently investigated, with only two relatively small studies thus far [12, 13].

One case‐control study (*n* = 215 CeAD cases) reported that 7% of individuals with CeAD had an autoimmune thyroid disease, compared to none in age/sex‐matched population controls. However, the proportion affected by AT versus Graves' disease, another autoimmune thyroid disease, was unclear in this study [13]. Another case‐control study (*n* = 29) found that 31% of CeAD cases had autoimmune thyroid disease, significantly higher than the 7% in those with non‐CeAD ischemic stroke [12]. Of note, most patients with autoimmune thyroid disease had AT (*n* = 7; 78%) as opposed to Graves' disease (*n* = 2; 22%) [12]. In general, previous studies examining the association between autoimmune thyroid disease and CeAD were limited by small sample sizes, including only nine to 15 patients with thyroid disease each, as well as limited capacity to account for confounding variables [12, 13], highlighting the need for further research.

Collectively, these immunologic and structural mechanisms suggest AT may contribute to CeAD through multiple pathways that impair arterial integrity. For instance, anti‐thyroid peroxidase and anti‐thyroglobulin antibodies could trigger endothelial dysfunction through cross‐reactivity [13, 27], while hypothyroidism‐induced increased intima‐media thickness [6], arterial stiffness [28], and increased collagen‐to‐elastin ratios may weaken vessel walls [29]. Given the relatively high prevalence of AT in the general population [3, 4] and the potential for CeAD to cause stroke, clarifying the potential association between AT and CeAD has important clinical implications.

Given the gaps in the existing research, the present study tested the hypothesis that AT is associated with an increased risk of CeAD. We used a retrospective cohort design to compare the risk of CeAD in adults with newly diagnosed AT to matched euthyroid controls, with a 3‐year follow‐up, and secondary analyses exploring CeAD cumulative incidence and risk of stroke.

## 2 Materials and Methods

### 2.1 Study Design

This study followed a registered protocol [30], examining data from the preceding 10 years, including patients meeting selection criteria from 2014 to 2021, with a query date of August 19, 2024. Data were obtained from the TriNetX (TriNetX LLC; Cambridge, MA, US) network, which included over 127 million patients attending 93 medical centers and their affiliated health care offices at the time of our query [31, 32]. This specific study used the NLP version of the US Research Network in TriNetX, a federated real‐world data resource, which is accessed via a secure web platform. This network derives data chiefly from electronic health records. Data originates from individual healthcare organizations that are generally large and/or academically affiliated in the US. However, the specific identities of these sites remain anonymous, and they are not disclosed to end users to comply with network data‐use agreements and to aid de‐identification. Data span demographics, diagnoses, laboratory and genomic results, medications, and procedures, which are harmonized to standardized terminologies for cohort building and analyses. This data resource may be searched using Current Procedural Terminology (CPT) codes and International Classification of Diseases, 10th Revision (ICD‐10) codes, which are automatically converted to 9th Revision as needed [31, 32], among other possible search terms. TriNetX regularly monitors conformance, consistency, and completeness of data quality [31, 32], with completeness of medication data at least 87% [33]. TriNetX adheres to the Health Insurance Portability and Accountability Act (HIPAA).

The University Hospitals Institutional Review Board (IRB; Cleveland, OH, US; STUDY20250071) considered the present study to represent “Not Human Subjects Research” due to its use of de‐identified data from the online TriNetX platform, thereby making the present study exempt from IRB review and waiving the requirement for consent.

**Figure 1:**
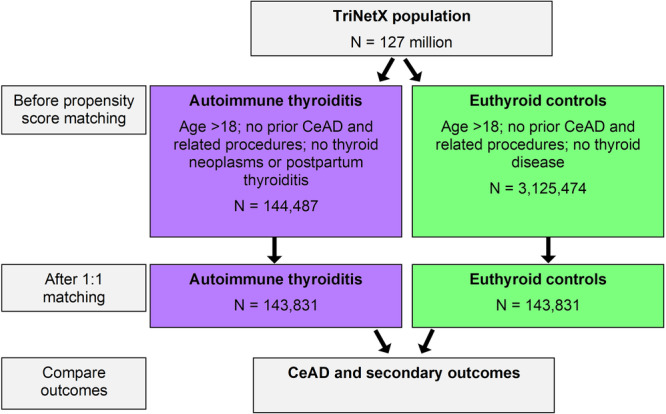
Study flow diagram. Patients with autoimmune thyroiditis and euthyroid controls, each meeting selection criteria, are selected from the overall TriNetX (TriNetX LLC, Cambridge, MA, US) population, forming two cohorts. These cohorts are then propensity matched, and outcomes are compared between them, including the primary outcome of cervical artery dissection (CeAD).

This study implemented natural language processing using software available within the TriNetX platform (Averbis, Freiburg im Breisgau, DE [32]). This feature extracts data from chart notes and test results [34, 35] and has been demonstrated to be adequately reliable and accurate compared to manual chart review [35, 36]. Natural language processing aimed to support our selection criteria and propensity matching strategy. A flowchart of the study design is shown in Figure 1.

### 2.2 Participants

#### 2.2.1 Eligibility Criteria

We included adults at least age 18, considering AT and CeAD typically affect adults [2, 37]. Individuals were divided into two cohorts: (1) Those with a new diagnosis of AT (ICD‐10: E06.3), and (2) those attending a general medical examination (ICD‐10: Z00.0) without thyroid disease. To improve data completeness, we required that individuals in both cohorts had a body mass index measurement within 3 years preceding or including the index date. To minimize loss to follow‐up, we required an additional health care visit within 3 years after the index date.

We excluded those with a history of CeAD and related procedures, and those with thyroid neoplasms and postpartum thyroiditis (Table S1). For those in the euthyroid cohort, we excluded patients having a diagnosis of thyroid disease, treatment used for thyroid disease, or a related laboratory test result suggestive of thyroid disease (Table S2).

Although thyroid function tests are available within the network, laboratory data are not uniformly complete, and such tests are not routinely obtained for all patients in real‐world care. We therefore avoided a test‐negative design that would have required a documented normal thyroid‐stimulating hormone level for inclusion. Requiring universal thyroid testing would have reduced the control cohort size and may have preferentially selected patients tested because of suspected thyroid dysfunction. Instead, euthyroid controls were defined by the absence of diagnoses, medications, procedures, or laboratory results consistent with thyroid disease.

### 2.3 Variables

We used propensity score matching to balance confounding variables between cohorts that have a known association with CeAD to minimize bias [38], considering variables within 3 years preceding and including the index date (Table S3). To minimize potential time‐related bias from differing years of cohort entry, we matched patients by their age at the index date and age at the time of our query (current age). This approach aimed to minimize confounding related to the increasing incidence of CeAD [2].

### 2.4 Primary Outcome

We identified diagnoses of CeAD using ICD‐10 codes (I77.71 [carotid artery dissection] and I77.74 [vertebral artery dissection]) along with natural language processing, over a 3‐year follow‐up window beginning the day after the index date of inclusion. A relatively long follow‐up window was preferred as the increase in risk of cardiovascular events in hypothyroidism is gradual [39]. Shorter follow‐up windows may therefore not capture time‐dependent cardiovascular effects of AT, whereas even longer follow‐up windows would diminish available sample size. A previous study using the TriNetX natural language processing dataset demonstrated adequate power for detecting associations between migraine and CeAD at 2 years' follow‐up, suggesting that 3 years would provide sufficient events for CeAD analysis [20]. Additionally, our team's previous TriNetX experience suggested that loss to follow‐up increased with extended follow‐up windows. Although we required one healthcare visit to minimize loss to follow‐up, requiring additional visits to enable even longer follow‐up could over‐represent patients with greater healthcare engagement or exclude those whose sole follow‐up visit was CeAD‐related. Accordingly, the 3‐year follow‐up weighed the need to examine the potential long‐term AT‐CeAD association against the risk of loss to follow‐up, the need to maintain statistical power, and minimize bias.

### 2.5 Secondary Outcomes

We explored the cumulative incidence of CeAD per cohort, and post‐matching RRs for carotid and vertebral artery dissection, as there were sufficient events (> 10 per each outcome). We also examined the post‐matching RR for stroke (ICD‐10: I60, I61, I62, and I63). We reported the proportion of patients in the AT cohort who received a thyroid disease‐related prescription (VA HS850 [thyroid modifiers], ATC H03 [thyroid therapy], or RxNorm 235479, 325521, or 10572 [porcine or beef thyroid]) and mean and median prescription/dispense count.

### 2.6 Statistical Methods

We used the TriNetX platform software to compare baseline characteristics, using standardized mean difference (SMD) with a threshold of > 0.1 representing residual imbalance. TriNetX uses logistic regression to calculate propensity scores using Python (scikit‐learn version 1.3 [Python Software Foundation, Delaware, US]). Propensity scores of 1 represent the greatest likelihood of being in the euthyroid cohort. Patients were matched 1:1 using greedy nearest‐neighbor matching and a caliper of 0.1 of the pooled standard deviations (SD). SMDs were calculated as the difference in means or proportions divided by the pooled SD. We reported the mean number of facts (e.g., any diagnosis, laboratory test, etc.) per patient as a descriptive measure of data density, and length of record and proportion of unknown demographics as measures of data completeness. The risk ratios (RRs) for CeAD and stroke were calculated by dividing the incidence in the AT cohort by the euthyroid cohort, using chi‐square tests to calculate *p*‐values. Risk ratios and their 95% confidence intervals were derived directly from 2 × 2 contingency tables. We plotted pre‐ and post‐matching propensity score densities, total incidence, and cumulative incidence of CeAD with 95% confidence intervals using R (version 4.2.2, Vienna, AT [40]) and ggplot2 [41]. We further assessed the success of propensity matching by deriving RRs for negative control outcomes unrelated to AT or CeAD [42]. Point estimates within a defined range (0.73 ≥ RR ≤ 1.38) suggested between‐cohort balance [43]. Negative control outcomes included administration of routine urinalysis (CPT: 1011224) and prescription of azithromycin (RxNorm: 18631). Follow‐up metrics, including mean, SD, and median duration, were obtained from de‐identified aggregate data exported from the TriNetX platform. TriNetX also provided data on patients lost to follow‐up, reported in 10‐day intervals over the 3‐year follow‐up window. We then plotted loss to follow‐up using R and ggplot2, showing the percentage of patients remaining per cohort over time by subtracting patient counts from the total cohort size.

### 2.7 Required Study Size

We calculated a total required sample size of 162,348 using G*Power (Kiel University, Germany) *z*‐tests, using a proportion between cohorts of 0.0003 versus 0.0007 for our primary outcome, using a power of 0.95, an allocation ratio of one, and a two‐tailed *α* error of 0.05 [2, 12].

## 3 Results

### 3.1 Participants

Before propensity matching, there were 144,487 patients in the AT cohort and 3,125,474 in the euthyroid controls cohort. After matching, AT and euthyroid controls cohorts each had 143,831 patients, with mean ages at index of (SD) 48.4 (15.8) and 48.9 (16.1) years, respectively. Both cohorts were predominantly female (80%). Before matching, patients in the AT cohort had a greater mean age at the index date and at the time of our query, a greater body mass index and proportion of overweight or obese individuals, and a greater proportion of females, among other differences (SMD > 0.1, Table 1). Following matching, all key variables were sufficiently balanced (SMD < 0.1, Table 1).

**Table 1:** Baseline characteristics before and after propensity score matching.

	Before matching			After matching		
Variable (*n* (%) or mean (SD))	Autoimmune thyroiditis	Euthyroid controls	SMD	Autoimmune thyroiditis	Euthyroid controls	SMD
*N*	144,487	3,125,474	NA	143,831	143,831	NA
Demographics						
Age at index	48.4 (15.8)	46.1 (17.2)	0.142	48.4 (15.8)	48.9 (16.1)	0.029
Age at query (current age)	54.6 (15.9)	52.6 (17.5)	0.122	54.6 (15.9)	55.1 (16.2)	0.029
Body mass index	29.3 (7.4)	28.9 (6.9)	0.668	29.3 (7.4)	29.4 (7.4)	0.003
Female	114,757 (80%)	1,534,986 (49%)	0.668	11,4742 (80%)	11,4891 (80%)	0.003
Male	17,491 (12%)	1,319,746 (43%)	0.725	17,491 (12%)	17,260 (12%)	0.005
Not Hispanic or Latino*	108,274 (75%)	2,302,433 (74%)	0.025	108,259 (75%)	105,976 (74%)	0.036
White*	103,591 (72%)	1,911,983 (62%)	0.222	103,578 (72%)	86,537 (60%)	0.252
Hispanic or Latino*	9245 (6%)	213,544 (7%)	0.018	9245 (6%)	10,924 (8%)	0.046
Other Race*	6468 (4%)	140,775 (5%)	0.002	6467 (4%)	6331 (4%)	0.005
Black or African American*	5409 (4%)	427,872 (14%)	0.360	5409 (4%)	21,679 (15%)	0.395
Asian*	5205 (4%)	129,394 (4%)	0.029	5205 (4%)	6243 (4%)	0.037
American Indian or Alaska Native*	395 (< 1%)	7840 (< 1%)	0.004	395 (< 1%)	384 (< 1%)	0.001
Native Hawaiian or Other Pacific Islander*	225 (< 1%)	7464 (< 1%)	0.019	224 (< 1%)	406 (< 1%)	0.027
Comorbidities						
Acute upper respiratory infections	21,443 (15%)	448,043 (14%)	0.013	21,440 (15%)	20,005 (14%)	0.028
Adverse socioeconomic and psychosocial circumstances	3925 (3%)	59,207 (2%)	0.055	3922 (3%)	3183 (2%)	0.033
Alpha‐1‐antitrypsin deficiency	52 (< 1%)	569 (< 1%)	0.011	52 (< 1%)	29 (< 1%)	0.010
Aortic aneurysm and dissection	945 (1%)	17,515 (1%)	0.012	944 (1%)	873 (1%)	0.006
Arterial fibromuscular dysplasia	73 (< 1%)	662 (< 1%)	0.015	73 (< 1%)	48 (< 1%)	0.008
Diabetes mellitus	21,903 (15%)	281,525 (9%)	0.189	21,893 (15%)	21,964 (15%)	0.001
Diseases of arteries, arterioles, and capillaries	8512 (6%)	96,221 (3%)	0.136	8497 (6%)	8072 (6%)	0.013
Ehlers‐Danlos syndromes	358 (< 1%)	1314 (< 1%)	0.054	346 (< 1%)	319 (< 1%)	0.004
Elevated white blood cell count	2842 (2%)	32,089 (1%)	0.077	2839 (2%)	2544 (2%)	0.015
Family history of ischemic heart disease	7608 (5%)	99,103 (3%)	0.104	7597 (5%)	6403 (4%)	0.039
Homocystinuria	287 (< 1%)	1262 (< 1%)	0.046	284 (< 1%)	267 (< 1%)	0.003
Hyperlipidemia	26,775 (19%)	531,425 (17%)	0.039	26,769 (19%)	26,308 (18%)	0.008
Hypertensive diseases	36,752 (26%)	830,983 (27%)	0.028	36,747 (26%)	36,672 (25%)	0.001
Migraine	11,483 (8%)	136,953 (4%)	0.148	11,471 (8%)	10,651 (7%)	0.021
Osteogenesis imperfecta	16 (< 1%)	209 (< 1%)	0.005	16 (< 1%)	10 (< 1%)	0.004
Other congenital syndromes affecting multiple systems	156 (< 1%)	2338 (< 1%)	0.011	156 (< 1%)	148 (< 1%)	0.002
Overweight and obesity	26,102 (18%)	434,333 (14%)	0.113	26,097 (18%)	25,928 (18%)	0.003
Substance use disorders	12,759 (9%)	303,265 (10%)	0.031	12,754 (9%)	11,722 (8%)	0.026
Tobacco use	4821 (3%)	112,144 (4%)	0.014	4819 (3%)	4095 (3%)	0.029
Medications						
Antihypertensives	6966 (5%)	184,108 (6%)	0.048	6965 (5%)	6652 (5%)	0.010
Antimigraine agents	7728 (5%)	90,428 (3%)	0.124	7722 (5%)	7386 (5%)	0.010
Beta blockers	20,388 (14%)	333,441 (11%)	0.104	20,379 (14%)	20,054 (14%)	0.007
Contraceptives, systemic	11,957 (8%)	217,996 (7%)	0.048	11,956 (8%)	10,771 (7%)	0.031
Quinolones	13,605 (9%)	241,674 (8%)	0.059	13,603 (9%)	13,183 (9%)	0.010

*Note:* Variables that were not matched, and reported for descriptive purposes (*).

Abbreviations: (NA), not applicable; (SD), standard deviation; (SMD). standardized mean difference.

### 3.2 Data Quality and Balance Diagnostics

The mean number of facts (i.e., diagnoses, lab tests, etc.) per patient per cohort was 2269 in the AT cohort and 1525 in the euthyroid controls cohort). Following matching, comparing those with AT versus euthyroid controls, the proportion of patients with unknown demographics were similar, including unknown ethnicity (18% vs. 19%; SMD = 0.011), unknown race (16% vs. 15%; SMD = 0.006), unknown gender (8% each; SMD = 0.002), and unknown age (0% each; SMD = 0). After matching, propensity score densities were observed to overlap closely, while SMD values for matched covariates were below the a priori threshold (< 0.1), indicating adequate balance of covariates (Figures S1 and S2). The mean follow‐up duration (SD) (AT: 955 [296]; euthyroid controls: 968 [279] days), median follow‐up duration (both cohorts: 1095 days), and loss to follow‐up were similar between cohorts (SMD = 0.045; Figure S3). Following matching, the risk of negative control outcomes was similar when comparing the AT cohort to euthyroid controls, per our a priori threshold (0.73 ≥ RR ≤ 1.38), further indicating that cohorts were successfully balanced. These included the RR for urinalysis [SD] (incidence: 23% vs. 27%; RR = 0.85 [0.84,0.86]) and azithromycin prescription (incidence: 12% vs. 13%; RR = 0.97 [0.95,0.99]). Together, these findings suggest there were negligible differences between cohorts regarding data density, data completeness, attrition, and covariate balance.

After matching, the geographic distribution of patients in the AT cohort spanned broad US regions, including the Northeast (39%), South (22%), West (20%), and Midwest (17%), with the remainder outside of the US or in US territories or in an unknown location. The euthyroid control cohort showed a similar pattern with most patients in the Northeast (37%), South (28%), Midwest (27%), and West (7%). Due to de‐identification requirements for the TriNetX network, geographic information is based on the headquarters of contributing healthcare organizations and categorized into regions mirroring the US census. This limits more granular spatial analyses, such as state or urban‐rural categories. Accordingly, these regional values are descriptive only and should be interpreted with caution. Figures illustrating the geographic distribution of patients are shown in Figure S4 and S5.

As a validity check, we compared thyroid‐stimulating hormone (TSH) values among patients with available laboratory data before matching. TSH levels were meaningfully greater and more variable in the AT cohort (mean 7.0, SD = 39.9) compared to controls (mean 1.7, SD = 0.8), with an SMD of 0.19. However, TSH test data in the 3‐year covariate assessment window were available in only 65% and 34% of the cohorts, respectively. Although our cohort definitions were based on the presence or absence of thyroid autoantibodies, rather than hormone levels, these findings indirectly support the face validity of the cohort definitions, considering TSH is typically greater among those with AT [37].

### 3.3 Primary Outcome

**Figure 2:**
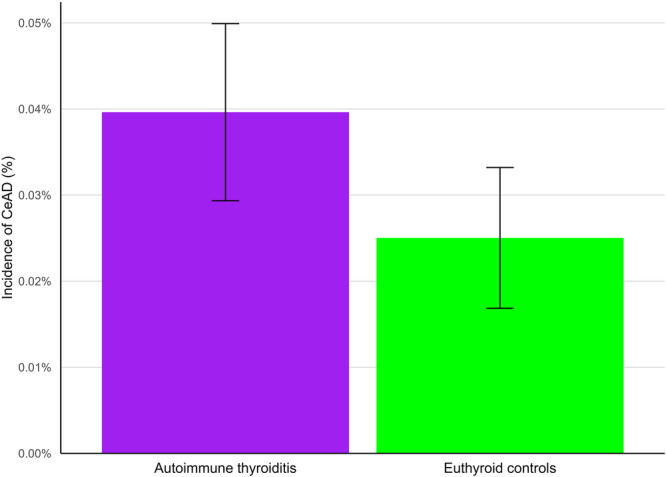
Incidence of cervical artery dissection (CeAD) per cohort after propensity matching. CeAD occurs in a smaller proportion of patients in the autoimmune thyroiditis cohort (purple) compared to euthyroid controls (green). While the 95% confidence intervals overlap slightly, there remains a statistically significant difference as indicated by the risk ratio and *p*‐value (Table 2).

The incidence and risk of CeAD were greater among the AT cohort compared to matched euthyroid controls [95% CI] (AT: 0.040%, euthyroid controls: 0.025%; RR 1.58 [1.04,2.40]; *p* = 0.029) (Figure 2 and Table 2). The incidence per 100,000 patients was 13.2 in the AT cohort and 8.3 in the euthyroid control cohort.

**Table 2:** Incidence and risk ratio of cervical artery dissection within 3 years following the index date.

	Before matching		After matching*	
	Autoimmune thyroiditis	Euthyroid controls	Autoimmune thyroiditis	Euthyroid controls
Number of patients	144,487	3,125,474	143,831	143,831
CeAD *n* (%)	57 (0.039%)	74 (0.024%)	57 (0.040%)	36 (0.025%)
CeAD *n* per 100,000 person‐years	13.1	7.9	13.2	8.3
CeAD risk ratio (95% CI); *p*‐value	1.66 (1.27, 2.17); *p* < 0.001	Reference	1.58 (1.04, 2.40); *p* = 0.029	Reference

Abbreviation: Cervical artery dissection (CeAD).

* Data used for the primary outcome.

### 3.4 Secondary Outcomes

**Figure 3:**
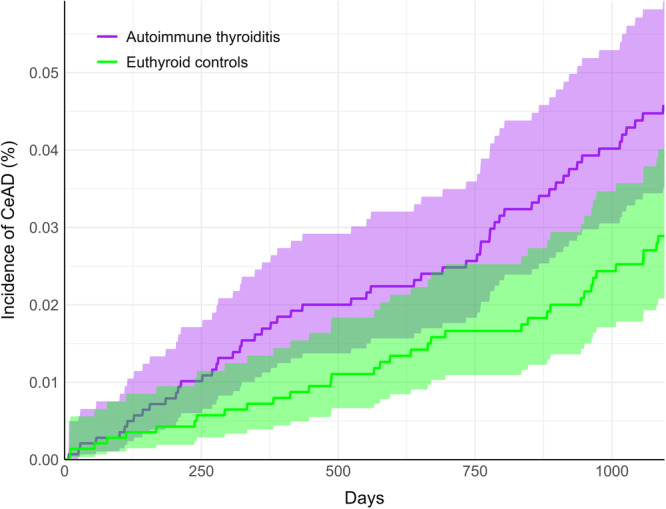
Cumulative incidence of cervical artery dissection (CeAD). The autoimmune thyroiditis cohort is shown in purple and euthyroid controls are shown in green, over the three‐year follow‐up window. Shaded regions show 95% confidence intervals.

A greater incidence of CeAD in the AT cohort relative to the euthyroid controls became apparent over increasing follow‐up (Figure 3). Although there was overlap between the 95% confidence interval regions for individual days, our hypothesis testing was based on the RR, which considers the entire follow‐up window and identified a significant AT‐CeAD association.

Comparing the AT cohort to euthyroid controls following matching, the incidence and risk of vertebral artery dissection were greater yet not statistically significant (32 vs. 25 patients; 0.022% vs. 0.017%; RR = 1.28 [0.76, 2.16]; *p* = 0.354). The incidence and risk of carotid artery dissection were significantly greater (28 vs. 12 patients; 0.019% vs. 0.008%; RR = 2.33 [1.19, 4.59]; *p* = 0.0114). The incidence and risk of stroke were also significantly greater in the AT cohort compared to euthyroid controls (3020 vs. 1806 patients; 2.1% vs. 1.3%; RR = 1.67 [1.58, 1.77,]; *p* < 0.001).

The proportion of patients who were prescribed thyroid medication during the 3‐year follow‐up was 62%, with a mean count of 7.9 prescriptions or dispenses (SD = 16.0; median = 4).

## 4 Discussion

This study suggests that adults with AT have a significantly increased risk of developing CeAD compared to matched euthyroid controls. Secondarily, results demonstrated a significantly increased risk of stroke and carotid artery dissection among those with AT. However, our study was not powered to detect differences in our secondary outcomes of individual subtypes of CeAD (carotid and vertebral artery dissection), which should be interpreted with some caution. While our primary point estimate revealed a modest AT‐CeAD association (RR = 1.58), the secondary positive association between AT and stroke supports its clinical relevance and meaningfulness, considering CeAD is a known etiology of stroke [1].

Any direct comparison of our findings to previous studies should be made cautiously due to differences in study demographics, data range, and selection criteria. However, the incidence of CeAD in the present study among those with AT was 13.2 per 100,000 person‐years, which exceeded a previous epidemiological estimate for CeAD in a US adult population of 8.9 per 100,000 person‐years [2]. Our observed incidence of CeAD among euthyroid controls was more comparable to this previous estimate (i.e., 8.3 *vs.* 8.9 per 100,000). Our female predominance in the AT cohort (80%) is corroborated by data from 2019, which similarly found that 76.2% of patients with AT were female [4]. Finally, the proportion of AT patients taking thyroid medication during follow‐up (62%) is comparable to the findings of a previous US study from 2019, which reported that the proportion of AT patients taking medication was 85% and progressively decreasing [4].

Our findings corroborate the two previous studies that identified a positive association between autoimmune thyroid disease and CeAD [12, 13]. While previous studies were case‐control designs, our cohort design allowed us to directly estimate risk, total incidence, and cumulative incidence. Additionally, a larger sample size, including 143,831 patients with AT, allowed us to robustly examine risk while controlling for several confounding variables, compared to past studies that included only up to 15 patients with autoimmune thyroid disease [12, 13]. Regardless, the repeated identification of a positive association across different study designs and populations further supports a meaningful association between AT and CeAD that is unlikely to be explained by comorbidities and factors that were propensity matched [14].

The pathophysiological effects of AT on the vasculature may account for the observed positive AT‐CeAD association. AT contributes to a low‐grade inflammation and deposition of antibodies in vessel walls that leads to endothelial dysfunction [6, 13]. One author group has hypothesized that autoimmune disorders such as AT contribute to low‐grade inflammation and deposition of antibodies in vessel walls, leading to endothelial dysfunction [13, 27]. In this model, autoimmune antibodies associated with AT, such as anti‐thyroid peroxidase or anti‐thyroglobulin antibodies, could cross‐react with proteins in the cervical arteries, triggering vessel dysfunction [13, 27]. Additionally, chronic inflammation among individuals with AT, including inflammatory mediators and cytokines, could contribute to microangiopathy or macroangiopathy [13, 27]. This hypothesis is not unique to CeAD, as anti‐thyroid antibodies have also been suspected of triggering other forms of vascular dysfunction, for instance, in Hashimoto's encephalopathy and moyamoya disease [44, 45]. However, we acknowledge that these mechanisms are hypothetical at this stage, as we are unaware of any pathology studies that have directly demonstrated immune complex deposition in the cervical arteries of patients with AT.

Other mechanisms could also account for the observed positive AT‐CeAD association. In animal studies, hypothyroidism has been shown to increase collagen and decrease elastic fibers in the aortic wall [29]. Additionally, hypothyroidism increases intima‐media thickness of the carotid artery in humans [6], and may cause increased vascular stiffness [28]. Considering our cumulative incidence revealed a relatively gradual and linear increase in CeAD, these factors may have time‐dependent effects.

While potential pathophysiological mechanisms linking AT and CeAD remain speculative and require histopathological confirmation, a convergence of clinical and epidemiological evidence strengthens their biological plausibility. First, thyroid autoimmunity has been implicated in diverse cerebrovascular conditions, including Hashimoto's encephalopathy and moyamoya disease [44, 45]. Second, hypothyroidism induces structural arterial changes, including increased carotid intima‐media thickness [6], increased arterial stiffness [28], and altered aortic wall composition with increased collagen and decreased elastic fibers [29]. Third, AT is an established risk factor for stroke [5] and multiple cardiovascular diseases [6, 7, 8, 9], including aortic and spontaneous coronary artery dissection [10, 11]. Fourth, emerging research suggests that other autoimmune conditions, namely, systemic lupus erythematosus, may increase CeAD risk [17]. Supported by the context of this prior research, our findings raise the possibility that endocrine and/or immune‐mediated components of AT may increase vulnerability to CeAD.

The present findings of a positive AT‐CeAD association may aid clinicians in identifying those at risk of CeAD, a condition that can be challenging to diagnose. Early symptoms of CeAD may include benign‐appearing features such as neck pain and headache, and the lag time to diagnosis is a mean of 9.2 days (SD = 12.2) [46]. While AT may represent a modest CeAD risk factor individually, its presence alongside characteristic CeAD symptoms and other risk factors (e.g., hypertension, pregnancy [14, 21]) may increase the index of suspicion for CeAD.

Additional research would be valuable to corroborate our findings and further clarify the AT‐CeAD association. Further large‐powered epidemiologic studies could better examine age subgroups or subtypes of CeAD with greater precision. Previous research has shown that the likelihood of cardiovascular sequelae associated with AT may vary depending on the level of treatment and hormone stability [7]. As such, it is necessary to examine whether the AT‐CeAD association varies depending on treatment, thyroid hormone stability, and antibody titer levels. Such studies may give additional insight into early detection and mitigation of CeAD in the AT population.

### 4.1 Strengths and Limitations

Strengths of this study include adherence to a registered protocol, a multidisciplinary author team, a large sample size, and a thorough propensity‐matching strategy. However, several limitations should be noted. The prevalence of AT and the approach towards its treatment vary globally [3]. Accordingly, our findings may not be generalizable to countries outside of the US. Our findings also do not apply to those with Graves' disease. Controlling for AT treatment approaches during follow‐up was not possible given the present design. There may be unmeasured confounding related to socioeconomic factors. The positive predictive value of ICD‐10 codes for CeAD is 90% [26], and it remains unclear whether our natural language processing strategy enhances this accuracy. As AT is a chronic disorder, it remains unclear whether the association with CeAD increases or plateaus over a follow‐up longer than 3 years. Because laboratory data were not uniformly available, and because our exposure of interest was autoimmune thyroiditis rather than thyroid hormone status, we did not require documented normal thyroid laboratory values for the euthyroid controls. Some misclassification is therefore possible. However, this limitation would be expected to bias our association with CeAD toward the null rather than augment it, as any unrecognized thyroid dysfunction among controls would make the two cohorts more similar. Accordingly, our estimates are likely conservative rather than being exaggerated by this potential bias.

## 5 Conclusions

The present study identified a statistically significant, positive association between AT and CeAD. This finding is likely clinically meaningful given the observed secondary outcome of a significant, positive association between AT and stroke. The relative increase in CeAD incidence among those with AT appears to be gradual and time‐dependent. These findings may aid clinicians in identifying patients at risk of CeAD, alongside other signs, symptoms, and risk factors. Further research is needed to clarify the variables that may mediate the AT‐CeAD association, such as medications used to treat AT and thyroid hormone and antibody levels.

## Author Contributions

Conceptualization: Robert J. Trager. Methodology and development of registered protocol: Robert J. Trager, Pratheek S. Makineni, and Debbie S. Wright. Software: Robert J. Trager. Formal analysis and investigation: Robert J. Trager, Pratheek S. Makineni, and Debbie S. Wright. Resources, data curation, and writing – original draft: Robert J. Trager. Writing – review and editing: Robert J. Trager, Pratheek S. Makineni, and Debbie S. Wright. Visualization, supervision and administration: Robert J. Trager. All authors have read and approved the final version of the manuscript. Robert J. Trager, the corresponding author and manuscript guarantor, had full access to all of the data in this study and takes complete responsibility for the integrity of the data and the accuracy of the data analysis.

## Ethics Statement

This study used de‐identified, anonymized data from TriNetX (TriNetX Inc., Cambridge, MA, US). At University Hospitals, the Clinical Research Center oversees access to TriNetX, provides user training, and ensures compliance with institutional data‐use policies. The lead study author, as a credentialed TriNetX user, directly developed the queries and performed analyses within the TriNetX platform. The University Hospitals Institutional Review Board (IRB; Cleveland, OH, US; STUDY20250071) considers the present study methods “Not Human Subjects Research”, therefore not requiring review board approval or patient consent.

## Conflicts of Interest

The authors declare that they have no known competing financial interests or personal relationships that could have appeared to influence the work reported in this paper.

## Transparency Statement

The lead author, Robert J. Trager, affirms that this manuscript is an honest, accurate, and transparent account of the study being reported; that no important aspects of the study have been omitted; and that any discrepancies from the study as planned (and, if relevant, registered) have been explained.

## Supporting information


**Figure S1:** Propensity score density plot. **Figure S2:** Covariate balance (Love) plot. **Figure S3:** A: This plot illustrates the percentage of patients remaining in each cohort throughout the duration of follow‐up, displaying the autoimmune thyroiditis cohort in purple and euthyroid controls in green. **Figure S4:** Geographic distribution of patients with autoimmune thyroiditis after matching. **Figure S5:** Geographic distribution of euthyroid patients after matching. **Table S1:** Exclusion criteria for both cohorts. **Table S2:** Exclusions for euthyroid cohort only variable. **Table S3:** Variables controlled for in propensity score matching.

## Data Availability

Minimal, aggregate, de‐identified datasets used for our primary outcome, cumulative incidence, and propensity score density plots are available in a Figshare repository (https://doi.org/10.6084/m9.figshare.27987086 [47]).
