# Supplementary material for: Association Between Autoimmune Thyroiditis and Cervical Artery Dissection: A Retrospective Cohort Study
Source: Health Sci Rep. 2026 Mar 22;9(3):e72161. doi: 10.1002/hsr2.72161 (PMC13097377; doi:10.1002/hsr2.72161)
Supplement: Supplementary file 1 — Figure S1: Propensity score density plot. Figure S2: Covariate balance (Love) plot. Figure S3: A: This plot illustrates the percentage of patients remaining in each cohort throughout the duration of follow‐up, displaying the autoimmune thyroiditis cohort in purple and euthyroid controls in green. Figure S4: Geographic distribution of patients with autoimmune thyroiditis after matching. Figure S5: Geographic distribution of euthyroid patients after matching. Table S1: Exclusion criteria for both cohorts. Table S2: Exclusions for euthyroid cohort only variable. Table S3: Variables controlled for in propensity score matching. [file HSR2-9-e72161-s001.pdf]

Supplemental File for: Association between autoimmune thyroiditis and cervical artery dissection: a retrospective cohort study

Table S1: Exclusion criteria for both cohorts

ICD-10 Codes	Definition	Window (days)
Diagnoses		
C73	Malignant neoplasm of thyroid gland	-∞ to 0
D09.3	Carcinoma in situ of thyroid and other endocrine glands	-∞ to 0
D34	Benign neoplasm of thyroid gland	-∞ to 0
D44.0	Neoplasm of uncertain behavior of thyroid gland	-∞ to 0
I60-I69 (ICD-10)	Cerebrovascular diseases	-∞ to 0
I77.71 (ICD-10)	Dissection of carotid artery	-∞ to 0
I77.74 (ICD-10)	Dissection of vertebral artery	-∞ to 0
I77.75 (ICD-10)	Dissection of other precerebral arteries	-∞ to 0
S15.0 (ICD-10)	Injury of carotid artery of neck	-∞ to 0
S15.0 (ICD-10)	Injury of vertebral artery	-∞ to 0
Z85.850 (ICD-10)	Personal history of malignant neoplasm of thyroid	-∞ to 0
090.5 (ICD-10)	Postpartum thyroiditis	-30 to 0
V00-Y99 (ICD-10)	External causes of morbidity (e.g., motor vehicle collisions, falls)	-30 to 0
Procedures and visits		
1022277 (CPT)	Transcatheter placement of extracranial vertebral artery stent(s), including radiologic supervision and interpretation, open or percutaneous	-∞ to 0
1022228 (CPT)	Transcatheter placement of intravascular stent(s), cervical carotid artery, open or percutaneous, including angioplasty, when performed, and radiological supervision and interpretation	-∞ to 0
35301 (CPT)	Thrombarterectomy, including patch graft, if performed; carotid, vertebral, subclavian, by neck incision	-∞ to 0
03QJ, 03QH, 03QN, 03QM, 03QL, 03QK (ICD-10-PCS)	Carotid artery repair	-∞ to 0
03QQ, 03QP (ICD-10-PCS)	Vertebral artery repair	-∞ to 0

Abbreviations: any preceding duration of time available in the patient's data (-∞);Current Procedural Terminology (CPT); International Classification of Diseases, 10th Revision (ICD-10); ICD-10 Procedure Coding System (ICD-10-PCS)

Table S2: Exclusions for euthyroid cohort only

Variable	Description and filter	Time window (days)
Diagnoses		
E00-E07 (ICD-10)	Disorders of thyroid gland	-∞ to 1095
R94.6 (ICD-10)	Abnormal results of thyroid function studies	-∞ to 1095
S19.84 (ICD-10)	Other specified injuries of thyroid gland	-∞ to 1095
T38.1 (ICD-10)	Poisoning by, adverse effect of and underdosing of thyroid hormones and substitutes	-∞ to 1095
T38.2 (ICD-10)	Poisoning by, adverse effect of and underdosing of antithyroid drugs	-∞ to 1095
090.5 (ICD-10)	Postpartum thyroiditis	-∞ to 1095
Laboratory tests		
LG11488-0*	Triiodothyronine, free (mass/volume) in serum, plasma, or blood. Filter: ≤259 pg/dL	-∞ to 1095
LG11488-0*	Triiodothyronine, free (mass/volume) in serum, plasma, or blood. Filter: ≥481 pg/dL	-∞ to 1095
LG11953-3*	Triiodothyronine (mass/volume) in serum, plasma, or blood. Filter: ≥ 181 ng/dL	-∞ to 1095
LG11953-3*	Triiodothyronine (mass/volume) in serum, plasma, or blood. Filter: ≤ 59 ng/dL	-∞ to 1095
LG12004-4*	Thyroxine.free [mass/volume] in serum, plasma, or blood: ≤ 0.8 ng/dL	-∞ to 1095
LG12004-4*	Thyroxine.free [mass/volume] in serum, plasma, or blood: ≥ 1.7 ng/dL	-∞ to 1095
LG359-6*	Thyroperoxidase Ab [units/volume] in serum, plasma, or blood: ≥ 34 [IU]/mL	-∞ to 1095
LG21864-0*	Thyroglobulin Ab [units/volume] in serum, plasma, or blood: ≥ 10 IU/mL	-∞ to 1095
5385-0 (LOINC)	Thyrotropin receptor Ab [units/volume] in serum: ≥ 1 IU/L	-∞ to 1095
9040*	Thyrotropin [units/volume] in serum, plasma, or blood. Filter: ≥ 4.1 m[IU]/L	-∞ to 1095
9041*	Thyroxine (T4) [mass/volume] in serum, plasma, or blood: ≤ 4.9 ug/dL	-∞ to 1095
9041*	Thyroxine (T4) [mass/volume] in serum, plasma, or blood: ≥ 12.1 ug/dL	-∞ to 1095
30166-3 (LOINC)	Thyroid stimulating immunoglobulins (actual/normal) in serum. Filter: ≥130% basal activity	-∞ to 1095
3013-0 (LOINC)	Thyroglobulin (mass/volume) in serum or plasma. Filter ≥ 4000 ng/dL	-∞ to 1095
3052-8 (LOINC)	Triiodothyronine (T3).reverse [mass/volume] in serum or plasma. Filter: ≥ 250 pg/mL	-∞ to 1095
Medications		
H03 (ATC)	Thyroid therapy	-∞ to 1095
HS850 (VA)	Thyroid modifiers	-∞ to 1095
10572 (RxNorm)	Thyroid (United States Pharmacopeia)	-∞ to 1095
235479 (RxNorm)	Thyroid, porcine	-∞ to 1095
325521 (RxNorm)	Thyroid, beef	-∞ to 1095
A9517 (HCPCS)	Iodine I-131 sodium iodide capsule(s), therapeutic, per millicurie	-∞ to 1095
V10XA (ATC)	Iodine (131I) compounds	-∞ to 1095
1546394 (RxNorm)	Iodine I-131	-∞ to 1095
Procedures		
1009025 (CPT)	Surgical procedures on the thyroid gland	-∞ to 1095
DGY5 (ICD-10-PCS)	Endocrine system, other radiation, thyroid	-∞ to 1095
DG05 (ICD-10-PCS)	Endocrine system, beam radiation, thyroid	-∞ to 1095
0GCK (ICD-10-PCS)	Endocrine system, extirpation, thyroid gland	-∞ to 1095
0G5K (ICD-10-PCS)	Endocrine system, destruction, thyroid gland	-∞ to 1095
DG15 (ICD-10-PCS)	Endocrine system, brachytherapy, thyroid	-∞ to 1095
0G9K (ICD-10-PCS)	Endocrine system, drainage, thyroid gland	-∞ to 1095
0GTG, 0GTH, 0GTJ, 0GTK (ICD-10-PCS)	Endocrine system, resection, thyroid gland, lobe, or isthmus	-∞ to 1095
0GBJ, 0GBH, 0GBJ (ICD-10-PCS)	Endocrine system, excision, thyroid gland or isthmus	-∞ to 1095
13619001 (SNOMED)	Thyroidectomy	-∞ to 1095
15463004 (SNOMED)	Operation on thyroid gland	-∞ to 1095
20470003 (SNOMED)	Destructive procedure on thyroid gland	-∞ to 1095
64291000052106 (SNOMED)	Radioactive iodine therapy	-∞ to 1095

Abbreviations: any preceding duration of time available in the patient's data (-∞), TriNetX curated code (*); Anatomical Therapeutic Chemical (ATC) Classification, Current Procedural Terminology (CPT); International Classification of Diseases, 10th Revision (ICD-10); ICD-10 Procedure Coding System (ICD-10-PCS); Logical Observation Identifiers Names and Codes (LOINC®); Systematized Nomenclature Of Medicine (SNOMED); Veterans Affairs National Drug File (VA)

Table S3: Variables controlled for in propensity score matching

Variable	Description	Association with CeAD	Reference(s)
Demographics	Age (index and current), sex	NA	
Diagnoses (ICD-10)			
E08-E13	Diabetes mellitus	⬇	[1–3]
E66	Overweight and obesity	⬇	[1]
E72.11	Hyperhomocysteinemia	⬆	[4]
E78.5	Hyperlipidemia, unspecified	⬇	[1, 2, 5]
E88.01	Alpha-1-antitrypsin deficiency	⬆	[6]
F10-F19	Mental and behavioral disorders due to psychoactive substance use	⬆	[7, 8]
D72.82	Elevated white blood cell count	⬆	[9]
G43	Migraine	⬆	[1]
J00-J06	Acute upper respiratory infections	⬆	[10, 11]
I10-I1A	Hypertensive diseases	⬆	[1]
I70-I79	Diseases of arteries, arterioles, and capillaries (includes aneurysms, arterial embolism, arteritis, and arterial fibromuscular dysplasia)	⬆	[12, 13]
I71	Aortic aneurysm and dissection	⬆	[13]
I77.3	Arterial fibromuscular dysplasia	⬆	[12]
Q78.0	Osteogenesis imperfecta	⬆	[12]
Q79.6	Ehlers-Danlos syndromes	⬆	[12]
Q87	Other specified congenital malformation syndromes affecting multiple systems (includes Marfan syndrome, Loeys Dietz syndrome, Alport syndrome, arterial tortuosity syndrome)	⬆	[12, 14]
Z55-Z65	Persons with potential health hazards related to socioeconomic and psychosocial circumstances	⬇	[10, 15]
Z72.0	Tobacco use	⬆	[1, 2]
Z82.4	Family history of ischemic heart disease and other diseases of the circulatory system	⬆	[1]
Labs/tests			
9083*	Body mass index (kg/m²)	⬇	[1]
Procedures/treatments			
AM400 (VA)	Quinolones (includes fluoroquinolones)	⬆	[16]
CN105 (VA)	Antimigraine agents (includes triptans)	⬆	[17]
CV100 (VA)	Beta blockers/related	⬇	[1]
CV490 (VA)	Antihypertensives, other	⬇	[1, 5]
HS200 (VA)	Contraceptives, systemic	⬆	[18]
Abbreviations: International Classification of Diseases, 10th Edition (ICD-10), Veterans Affairs National Drug File (VA), custom curated TriNetX code (*), positive association (⬆), negative association (⬇)			

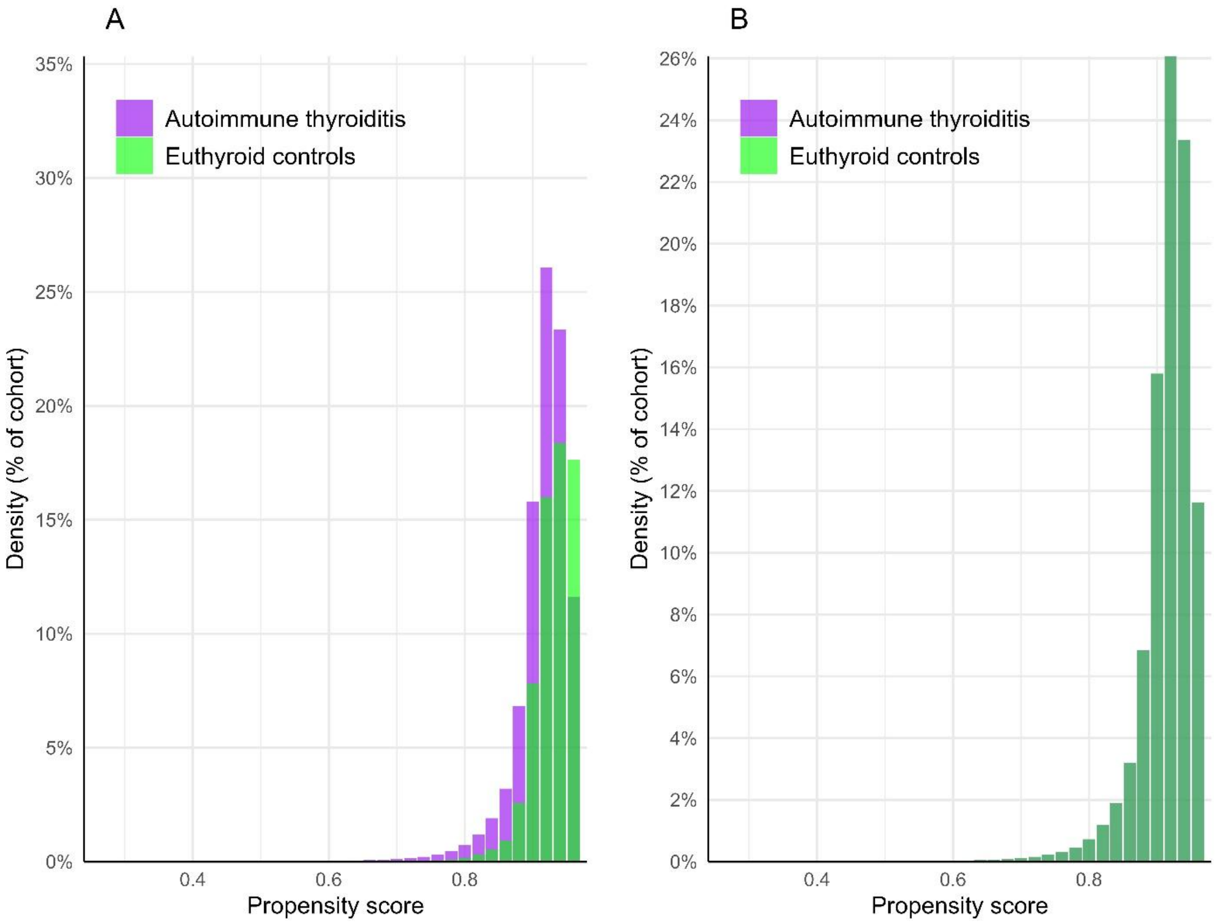

Figure S1: Propensity score density plot. Propensity scores before (A) and after (B) matching. The purple bars represent the autoimmune thyroiditis (AT) cohort while the gray bars represent euthyroid controls. After matching, propensity score densities overlap tightly suggesting that covariates are adequately balanced.

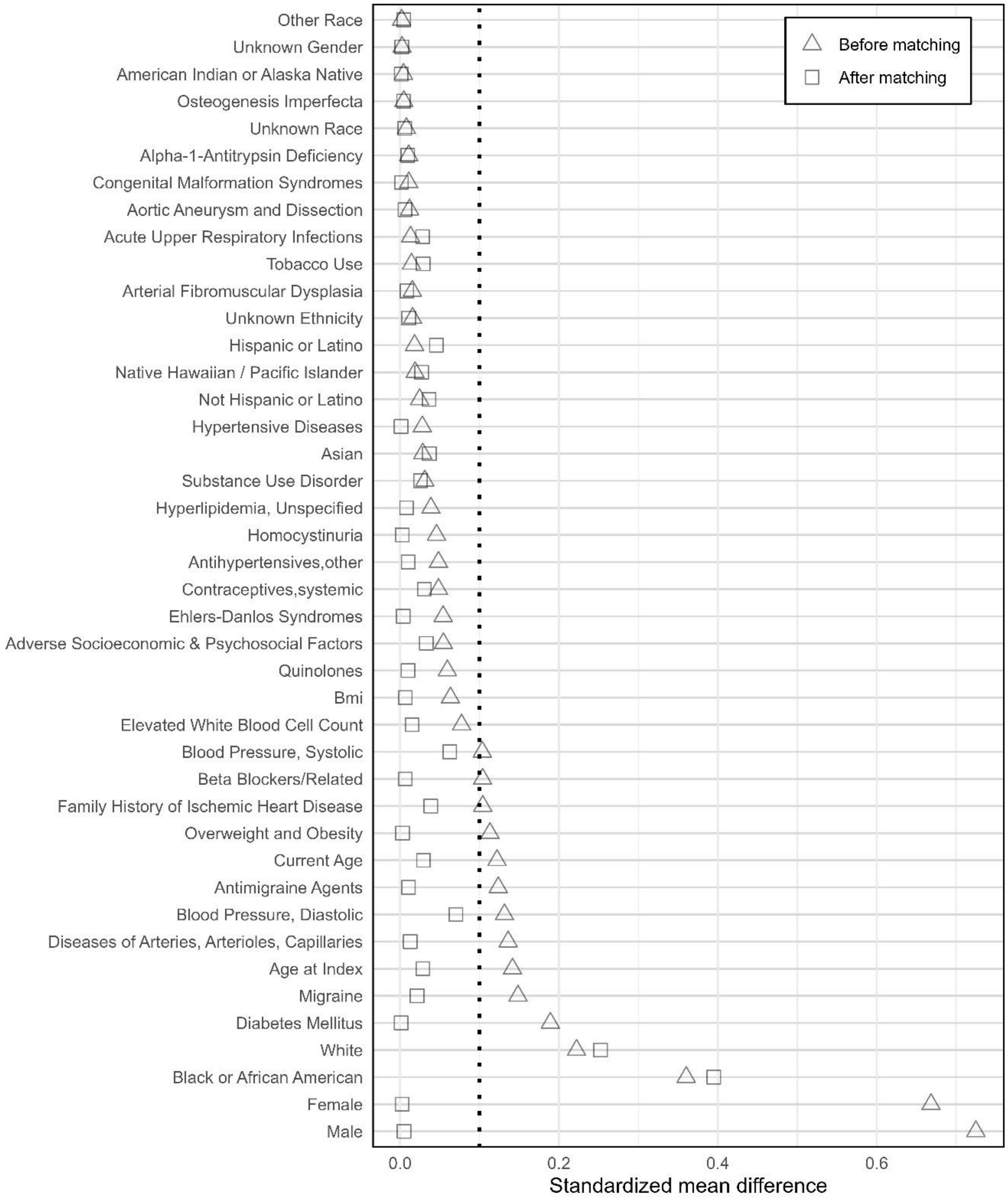

Figure S2: Covariate balance (Love) plot. This illustrates the standardized mean differences (SMDs) between cohorts before and after propensity score matching. The vertical dashed line at SMD=0.1 represents the threshold for acceptable covariate balance [19, 20]. Triangles show SMDs before matching per covariate, while squares show SMDs after matching. This plot demonstrates the improvement in covariate balance through propensity score matching, with all covariates ultimately having adequate balance after matching. Abbreviations: body mass index (bmi). Plot created by Robert J. Trager using R and R studio (version 4.2.2, Vienna, AT [21]) and the ggplot2 package [22]

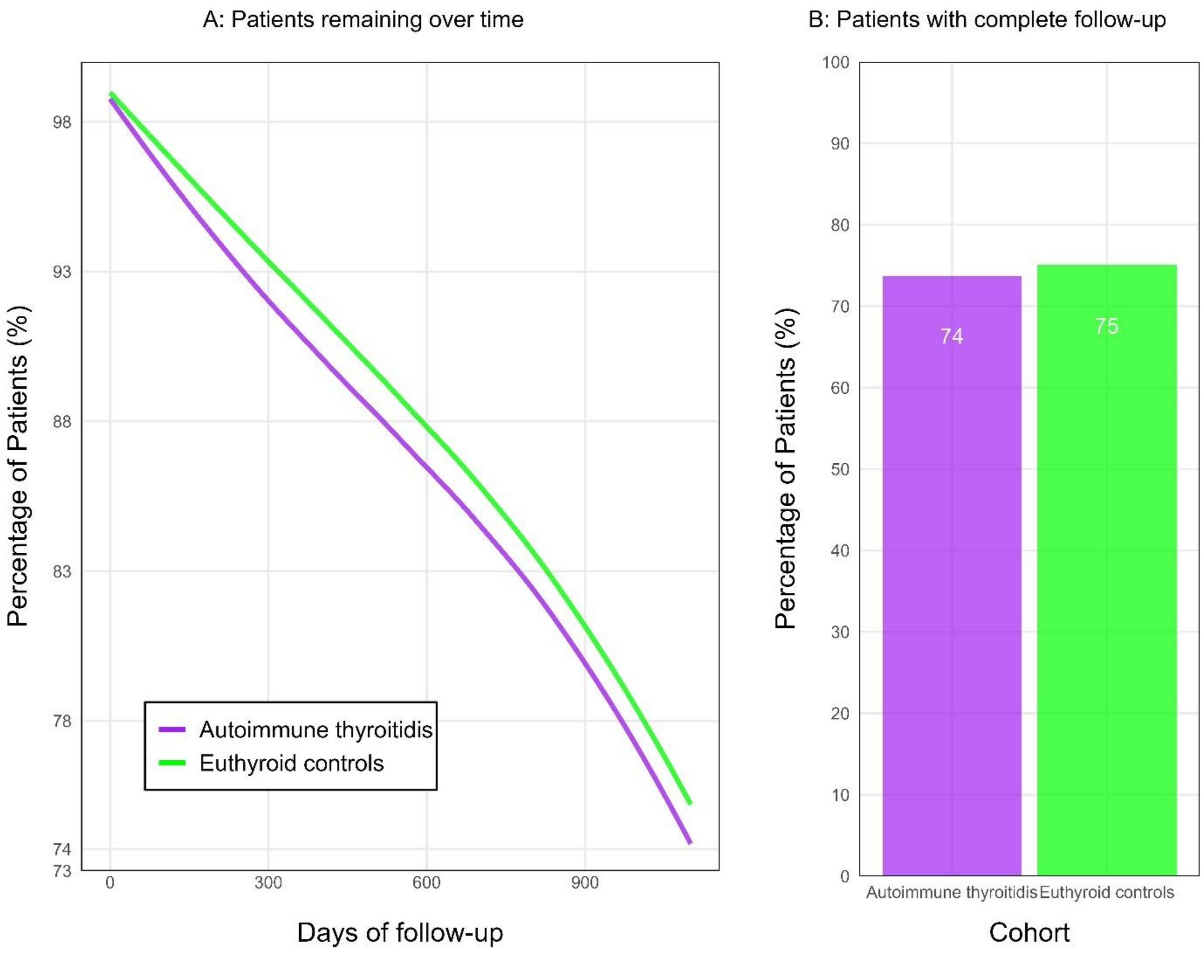

Figure S3: A: This plot illustrates the percentage of patients remaining in each cohort throughout the duration of follow-up, displaying the autoimmune thyroiditis cohort in purple and euthyroid controls in green. This plot incorporates locally estimated scatterplot smoothing. B: The bar plot displays the percentage of patients who remained in each cohort for at least the maximum follow-up time available (i.e., autoimmune thyroiditis: 74%; euthyroid controls: 75%; standardized mean difference=0.045). Both plots were created by Robert J. Trager using R and R studio (version 4.2.2, Vienna, AT [21]) and the ggplot2 package [22].

US Regions	Patients	Percent
Northeast	56,475	39%
Midwest	24,014	17%
South	31,476	22%
West	29,003	20%

Ex-US Regions	Patients	Percent
Ex-US	2,574	2%

Other Regions	Patients	Percent
Unknown	945	<1%

Patient location is determined by location of HCO headquarters

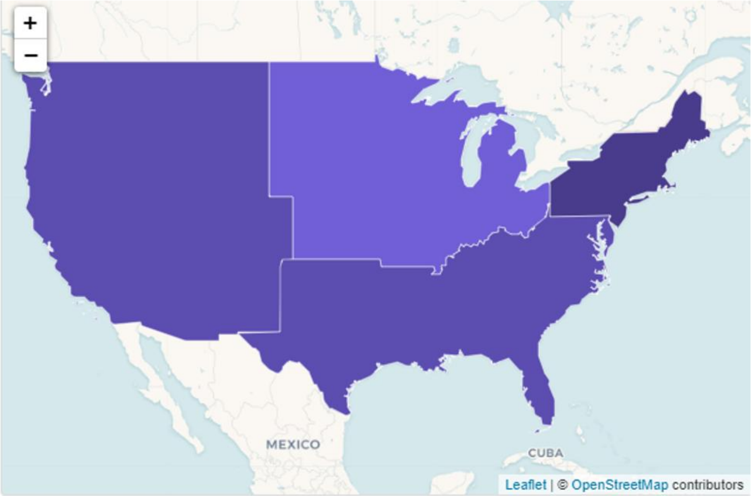

Figure S4: Geographic distribution of patients with autoimmune thyroiditis after matching. For purposes of de-identification, regions are aggregated into broad zones: Northeast, Midwest, South, West, Ex-US (which may include US territories), and Unknown. Darker purple shading indicates higher patient representation. Although not depicted, Alaska and Hawaii are included in the West region.

US Regions	Patients	Percent
Northeast	1,170,102	37%
Midwest	839,254	27%
South	869,196	28%
West	223,128	7%

Ex-US Regions	Patients	Percent
Ex-US	17,796	<1%

Other Regions	Patients	Percent
Unknown	5,998	<1%

Patient location is determined by location of HCO headquarters

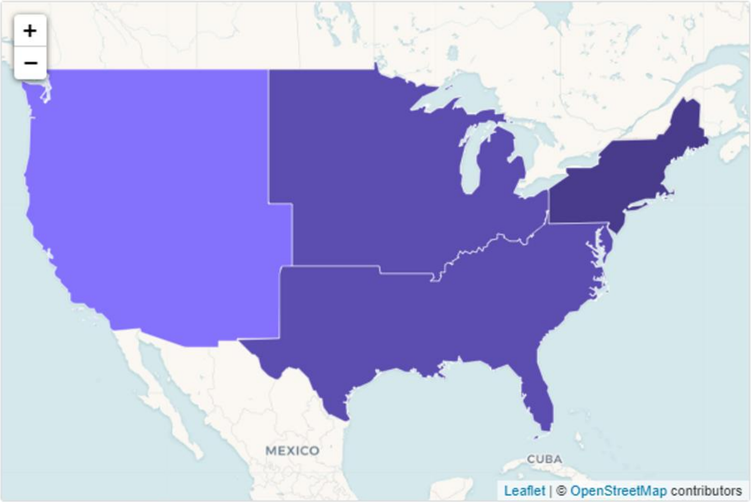

Figure S5: Geographic distribution of euthyroid patients after matching. For purposes of de-identification, regions are aggregated into broad zones: Northeast, Midwest, South, West, Ex-US (which may include US territories), and Unknown. Darker purple shading indicates higher patient representation. Although not depicted, Alaska and Hawaii are included in the West region.

References

1. Del Zotto E, Grassi M, Zedde M, Zini A, Bersano A, Gandolfo C, Silvestrelli G, Baracchini C, Cerrato P, Lodigiani C, Marcheselli S, Paciaroni M, Spalloni A, Cappellari M, Del Sette M, Cavallini A, Lotti EM, Delodovici ML, Gentile M, Magoni M, Padroni M, Azzini C, Calloni MV, Giorli E, Braga M, La Spina P, Melis F, Tassi R, Terruso V, Calabrò RS, Piras V, Giossi A, Sanguigni S, Zanferrari C, Mannino M, Colombo I, Dallocchio C, Nencini P, Bignamini V, Adami A, Bella R, Pascarella R, Keser Z, Pezzini A, Italian Project on Stroke in Young Adults-Cervical Artery Dissection, (IPSYS CeAD) Research Group (2023) Risk Profile of Patients with Spontaneous Cervical Artery Dissection. Ann Neurol 94:585–595. https://doi.org/10.1002/ana.26717

2. Abdelnour LH, Abdalla ME, Elhassan S, Kheirelseid EAH (2022) Meta-analysis of cardiovascular risk profile of stroke secondary to spontaneous cervical artery dissection compared to ischemic stroke of other causes. Health Sci Rev 5:100058. https://doi.org/10.1016/j.hsr.2022.100058

3. Engelter ST, Grond-Ginsbach C, Metso TM, Metso AJ, Kloss M, Debette S, Leys D, Grau A, Dallongeville J, Bodenant M, Samson Y, Caso V, Pezzini A, Bonati LH, Thijs V, Gensicke H, Martin JJ, Bersano A, Touzé E, Tatlisumak T, Lyrer PA, Brandt T, Cervical Artery Dissection and Ischemic Stroke Patients Study Group (2013) Cervical artery dissection: trauma and other potential mechanical trigger events. Neurology 80:1950–1957. https://doi.org/10.1212/WNL.0b013e318293e2eb

4. Gallai V, Caso V, Paciaroni M, Cardaioli G, Arning E, Bottiglieri T, Parnetti L (2001) Mild Hyperhomocyst(e)inemia. Stroke 32:714–718. https://doi.org/10.1161/01.STR.32.3.714

5. Abdelnour LH, Abdalla ME, Elhassan S, Kheirelseid EAH (2022) Diabetes, hypertension, smoking, and hyperlipidemia as risk factors for spontaneous cervical artery dissection: Meta-analysis of case-control studies. Curr J Neurol 21:183–193. https://doi.org/10.18502/cjn.v21i3.11112

6. Pezzini A, Magoni M, Corda L, Pini L, Medicina D, Crispino M, Pavia M, Padovani A, Grassi V (2002) Alpha-1-antitrypsin deficiency-associated cervical artery dissection: report of three cases. Eur Neurol 47:201–204. https://doi.org/10.1159/000057899

7. Hori S, Hori E, Umemura K, Shibata T, Okamoto S, Kubo M, Horie Y, Kuroda S (2020) Anatomical Variations of Vertebrobasilar Artery are Closely Related to the Occurrence of Vertebral Artery Dissection—An MR Angiography Study. J Stroke Cerebrovasc Dis 29:104636. https://doi.org/10.1016/j.jstrokecerebrovasdis.2020.104636

8. Smith WS, Johnston SC, Skalabrin EJ, Weaver M, Azari P, Albers GW, Gress DR (2003) Spinal manipulative therapy is an independent risk factor for vertebral artery dissection. Neurology 60:1424–1428. https://doi.org/10.1212/01.WNL.0000063305.61050.E6

9. Grond-Ginsbach C, Giossi A, Aksay SS, Engelter ST, Lyrer PA, Metso TM, Metso AJ, Tatlisumak T, Debette S, Leys D, Thijs V, Bersano A, Abboud S, Kloss M, Lichy C, Grau A, Pezzini A, Touzé E, Group C (2013) Elevated peripheral leukocyte counts in acute cervical artery dissection. Eur J Neurol 20:1405–1410. https://doi.org/10.1111/ene.12201

10. Grau AJ, Brandt T, Buggle F, Orberk E, Mytilineos J, Werle E, Conradt C, Krause M, Winter R, Hacke W (1999) Association of Cervical Artery Dissection With Recent Infection. Arch Neurol 56:851–856. https://doi.org/10.1001/archneur.56.7.851

11. Witsch J, Rutrick SB, Lansdale KN, Seitz A, Kamel H, Parikh NS, Segal AZ, Mir SA, Murthy SB, Niogi SN, Gaudino M, Girardi LN, Kim J, Devereux RB, Roman MJ, Iadecola C, Kasner SE, Zhang C, Merkler AE (2023) Influenza-Like Illness as a Short-Term Risk Factor for Arterial Dissection. Stroke 54:e66–e68. https://doi.org/10.1161/STROKEAHA.122.042367

12. Debette S (2014) Pathophysiology and risk factors of cervical artery dissection: what have we learnt from large hospital-based cohorts? Curr Opin Neurol 27:20–28. https://doi.org/10.1097/WCO.0000000000000056

13. Witsch J, Mir SA, Parikh NS, Murthy SB, Kamel H, Navi BB, Segal AZ, Fink ME, Rutrick SB, Safford MM, Narula N, Goyal P, Gaudino M, Girardi LN, Devereux RB, Roman MJ, Zhang C, Merkler AE (2021) Association Between Cervical Artery Dissection and Aortic Dissection. Circulation 144:840–842. https://doi.org/10.1161/CIRCULATIONAHA.121.055274

14. Traenka C, Kloss M, Strom T, Lyrer P, Brandt T, Bonati LH, Grond-Ginsbach C, Engelter S (2019) Rare genetic variants in patients with cervical artery dissection. Eur Stroke J 4:355–362. https://doi.org/10.1177/2396987319861869

15. Kellert L, Grau A, Pezzini A, Debette S, Leys D, Caso V, Thijs VN, Bersano A, Touzé E, Tatlisumak T, Traenka C, Lyrer PA, Engelter ST, Metso TM, Grond-Ginsbach C, Kloss M, Cervical Artery Dissection and Ischemic Stroke Patients (CADISP)-Study Group (2018) University education and cervical artery dissection. J Neurol 265:1065–1070. https://doi.org/10.1007/s00415-018-8798-7

16. Zotto ED, Pezzini A (2019) Use of fluoroquinolones and the risk of spontaneous cervical artery dissection. Eur J Neurol 26:1028–1031. https://doi.org/10.1111/ene.13917

17. Roberto G, Piccinni C, D'Alessandro R, Poluzzi E (2014) Triptans and serious adverse vascular events: Data mining of the FDA Adverse Event Reporting System database. Cephalalgia 34:5–13. https://doi.org/10.1177/0333102413499649

18. D'Anglejan-Chatillon J, Ribeiro V, Mas J l., Youl B d., Bousser M g. (1989) Migraine - A Risk Factor for Dissection of Cervical Arteries. Headache J Head Face Pain 29:560–561. https://doi.org/10.1111/j.1526-4610.1989.hed2909560.x

19. Austin PC (2009) Balance diagnostics for comparing the distribution of baseline covariates between treatment groups in propensity-score matched samples. Stat Med 28:3083–3107. https://doi.org/10.1002/sim.3697

20. Stuart EA, Lee BK, Leacy FP (2013) Prognostic score–based balance measures can be a useful diagnostic for propensity score methods in comparative effectiveness research. J Clin Epidemiol 66:S84-S90.e1. https://doi.org/10.1016/j.jclinepi.2013.01.013

21. R Core Team (2022) R: A Language and Environment for Statistical Computing. R Foundation for Statistical Computing, Vienna, Austria

22. Wickham H (2016) ggplot2: Elegant Graphics for Data Analysis. Springer-Verlag New York
